# Attitudes of Nurses and Hospitalized Patients about the Rights of Psychiatric Clients

**Published:** 2018-10

**Authors:** Sedigheh Ebrahimi, Easa Salehi Dehno

**Affiliations:** Department of Medical Ethics, Shiraz University of Medical Sciences, Shiraz, Iran.

**Keywords:** *Clients*, *Healthcare*, *Nurse*, *Psychiatry*, *Patients' Rights*

## Abstract

**Objective:** In recent years, protecting the rights of hospitalized psychiatric clients became a key issue in relationship-building and collaborative caretaking. Despite its importance, a few researches have been conducted on assessing the attitudes and expectations of clients and nurses about this issue. This study aimed to compare the nurses and psychiatric clients’ attitudes towards rights of hospitalized clients.

**Method** **:** In this cross sectional descriptive study, 60 nurses, and 100 clients, who were admitted to various wards of a psychiatric hospital in Shiraz, were included. Data were collected using 2 questionnaires that were designed for nurses (22 questions) and patients (29 questions) about the rights of patients. Data were analyzed by descriptive and inferential statistics.

**Results: **The results revealed that most of nurses (55%) and clients (64%) agreed with active participation of the clients in healthcare decision-making. More than half of the clients agreed with the possibility of refusal/withdrawal of the proposed treatment or leaving the hospital despite medical advice. Only 38.3% of nurses agreed with those rules in some clauses (P-value < 0.001).

**Conclusion: **To protect the rights of mentally ill clients, their family, and the society, we should identify weaknesses and shortcomings of the basic rights of this group and make suggestions for their improvement. A legal bill, which covers the rights of mentally ill clients, could be a turning point for improvement of the quality of care as well as increasing clients' satisfaction.

Human beings are creatures who have biological, mental, sociocultural, and spiritual needs and have certain rights during healthy and ill conditions. In recent decades, the issue of clients' rights has been of great interest. Clients' rights is defined as comprehensive healthcare providers’ assignments toward the protection of the delivery of health services to the people, especially in a specific situation (1, 2). This concept often becomes troublesome when applied to psychiatric clients requiring medical intervention (3, 4). These clients, despite their ability to make decisions about their life and future, may be deprived of freedom by compulsory admissions in mental health centers (5). 

The provision of appropriate care for these clients is a complex process with several barriers. Some of these barriers exist at the policy level, such as lack of a legal bill which covers the needs of these clients, and some other barriers are at the health system level, including low budget, shortage of inpatient beds, and inadequate knowledge of staff (6). 

The changing expectations of people toward their rights, with increasing levels of education and awareness, has led to an increasing clients’ expectation of their rights. So providers need for more knowledge and nobility of the healthcare delivery system to improve the quality of care. The specific situation of mentally ill clients and the importance? Of violation to their rights have gained the attention of healthcare providers in the formulation of healthcare legislation and policy, guidelines, standards, and trainings. Therefore, healthcare professionals, especially in the field of psychiatry, should be aware of the basic rights of these clients. This awareness can play an important role in providing satisfaction and improving the treatment plans (1, 2, 7 and 8). 

The aim of this study was to identify the differences between nurses and clients hospitalized in a general psychiatric hospital in Shiraz, Iran, with respect to their attitude towards multiple facets of psychiatric clients’ rights. The result of this study can provide the opportunity to recognize the weaknesses and shortcomings of care services to mitigate these problems so that clients would benefit more from their treatment plans and be exposed to less harm.

## Materials and Methods

This cross sectional descriptive study was conducted among nurses and hospitalized clients in a psychiatric hospital associated with Shiraz University of Medical Sciences in Iran. A total of 60 staff nurses and 100 clients were enrolled using a convenience sampling method. In the clients group, those who were selected by the physician or nurse in charge who received instruction by the researcher were recruited. The criteria for inclusion were a psychiatric diagnosis, being discharged after more than 3 days of inpatient care, willingness to participate, and being able to cooperate. 

Two separate questionnaires that had previously been established as valid and reliable were used to gather the required information. These questionnaires were previously developed by Abbasi et al. (2009) based on previously published literature, patient rights charter approved by the Ministry of Health and Medical Education of Iran (MOHME), and the legal bases for inpatient mental healthcare suggested by the World Health Organization (WHO) (7). 

In this study, the clarity of the questionnaire was approved by a group of experts in the field of medical ethics and psychiatry. The first section of both questionnaires was related to demographic information. Nurses’ attitude questionnaire comprised of 22 items related to the rights of clients in two parts (A and B). “A” shows the attitude of a nurse toward the importance of clients' charter and “B” shows their attitude towards the feasibility and practical capability of each option. In the next 10 questions, the individual's attitudes toward the necessity of some aspects of clients' rights were questioned. Clients’ questionnaire includes 29 questions concerning the patient’s attitudes toward the necessity of the clients’ rights. Both clients and nurses give their views on each question by giving a score, from 1 (strongly disagree) to 5 (strongly agree). 

Before completion of the questionnaires, a brief oral description about the study aim was provided. Informed verbal consents were taken from the study participants. They were also given full authority to withdraw from the study at any time during the study. The questionnaires were also completed anonymously to maintain confidentiality. 

Data were analyzed using descriptive and inferential statistics, including frequency tables and mean± standard deviation (SD), with the χ2 test through SPSS Version 19.0. A P-value of ≤ 0.05 was considered as statistically significant.

## Results

A total of 60 staff nurses and 100 clients participated in this study.

Table 1 displays the demographic characteristics of the nurses and the clients who had completed the questionnaires.

It was the first hospitalization for 53 clients (53%) and the second or more hospitalizations for 47 (47%) clients. More than half of the nurses (78.3%) agreed with the necessity of informing the mentally ill clients about their rights at the time of admission in the medical center. Regarding the active participation in all decision-making about diagnostic evaluations and treatment of the mentally ill clients, there was some disagreement between clients and nurses in a way that 55% of the nurses (n = 33) and 64% of the clients (n = 64) agreed to this case (P = 0.25). Only 40% of the nurses (n = 24) agreed on the applicability of this clause in the clinical practice. 

Considering the decision-making of the mentally ill clients on the withdrawal of treatment against the advice of the medical team, difference existed between the two groups, as 38.3% (n = 23) of the nurses and 73% (n = 73) of clients agreed about this item. Only 38.3% (n = 23) of the nurses had positive views about the applicability of this item. In the case of the possibility of clients' refusal of the proposed treatment recommended by the medical staff, 50% of the nurses and 73% of the clients agreed by giving this right to the patient. A total of 48.3% of the nurses and 74% of the clients agreed with the psychiatric clients leaving the hospital despite the advice of the medical team. Also, 53 nurses (88.3%) and 74 clients (74%) agreed about the right of the patient or his/her surrogate decision-makers to ask for the responses of the medical team to the questions about the chances of recovering from the disease. The attitudes of nurses and clients were compared in Table2.

## Discussion

WHO has emphasized on actions related to the following rules to ensure the protection of the rights of the persons with mental disorders: informed consent to treatment, confidentiality, least restrictive environment in delivering care, and avoidance of restraint and seclusion when possible, voluntary and involuntary hospitalization and treatment procedures, the presence of “dangerousness” and “need to treatment” as criteria for forced hospitalization, discharge procedures, and protection of user property"(9). 

The present study revealed that most of the clients and nurses agreed on the necessity of involuntary admission of the clients in the following situations: when a patient was not able to take care of his/her needs due to psychotic condition, after a suicidal attempt, or because they were seen as a danger to others. No significant differences were observed between the 2 groups in these cases. 

The result of a survey by Gardner et al. in 1999 indicated that clients who did not believe the need for hospitalization, reported more perceived coercion at admission. After the treatment period, however, 52% of them believed that their admission was necessary and useful. Gardner believed that most of clients will be aware of their need of hospitalization even if they initially refuse it (10). Kane et al., in a study performed in 1983, found significant positive changes in clients’ attitudes about the basic need for involuntary treatment and hospitalization they had initially resisted. Clients achieving remission of symptoms were most likely to have a positive attitude (5). 

The results of our study showed that more than half of the clients believed that for the mentally ill clients, there should be the possibility of withdrawing treatment, the possibility of leaving the hospital, and the possibility of treatment rejection against the advices of the medical staff. However, less than half of the nurses agreed with these items of perceived clients’ rights. 

The differences between the views of nurses and clients may be due to conflicts in the way each group values the clients’ rights and need for treatments. 

Regarding the situations that justify the confinement of clients in locked rooms and using physical coercion, there were significant differences between the 2 groups (P <0.001). The statistics suggest that clients are very concerned with the maintaining of their dignity during staying in hospital. 

Previous surveys showed that clients who had a compulsory admission to hospital and even mentioned it necessary in special conditions did not agree with the locked rooms and limitations of physical activity as well as the use of physical force, indicating that clients want to be treated with respect (8, 11). 

In this study, we surveyed privacy rights in 3 situations that compromised confidentiality. There were no significant differences between the groups in providing information concerning mental illness to the patient’s mate (P = 0.43) and in the case of passing on information in response to a query of the courts and legal authorities and to the patient’s employer, even without the patient's consent (P = 0.63). In fact, they believed that there is no need for keeping privacy in these cases. The literature revealed no significant differences between the staff and clients regarding attitudes towards clients’ rights to obtain information about their illness and treatment and also their right to refuse treatment (12). The providers of health services, especially medical teams, should be aware of the clients' rights and respect their choices and decisions (13).

In Iran, due to lack of clear and standard legislations in mental health, MOHME has introduced a draft of bill of rights in the context of treatment of clients with severe and chronic mental illness, which was regulated and standardized. Setting more complete and comprehensive charter on the one hand and adequate education of the medical team and the disclosure of clients' rights to them on the other, can help reach our ultimate goal of this research, which is helping to increase the satisfaction level of clients hospitalized in psychiatric hospitals. 

**Table 1:** Demographic Characteristics of the Clients and Nurses

**Variable**	**Nurses**	**Clients**
	**N (%)**	**N (%)**
Sex Male Female	35 (53.3) 25(41.7)	62(62) 38(38)
Age < 30 yrs. 30-50 yrs. > 50 yrs.	48(80) 12(20)	42(42) 45(45) 13(13)
Experiences of nurses in psychiatric ward (yrs.) < 5 yrs. > 5 yrs.	41(68.3%) 19(31.7%)	
Education Bachelor's degrees Master 's degrees Associate degree High school Diploma low literacy (not completed school years)	55(91.7 %) 5(8.3%)	2% 4% 21% 73%

## Limitation

Limitation of the study was the assessing the participants’ attitude by a questionnaire from a local hospital, may reveal different outcomes with the attitude in professional career.

## Conclusion

The differences in attitudes toward some aspects of clients' rights in the present study highlighted the importance of considering clients’ expectations regarding their rights. Each psychiatric patient has individual and social rights which must be considered by the healthcare providers. All clients are equally entitled to these rights without any discrimination and they should be expressed and guaranteed by law in the form of mental health statement of rights.

**Table 2:** The Attitudes of Clients and Nurses toward the Rights of Hospitalized Psychiatric Clients

	**Necessity**		**P-value**
	**Nurse**	**Patient**	
	**%(N)**	**%(N)**	
Patient should receive service regardless of race, language, religion, sex, physical or mental disability, socioeconomic status, etc.	86.7 (52)	72 (72)	0.037
The patient should have the right to access customer information services and access all services that are provided in the hospital.	88.3 (53)	72 (72)	0.016
Psychiatric patients should have the right to appropriate medical, psychosocial, and rehabilitative care, treatment, and training as soon as possible.	90 (54)	73 (73)	0.01
Patients should have the right to access the medical team during hospitalization.	81.7 (49)	75 (75)	0.000
Patients should have the right to access the medical team after being discharged from the hospital.	80 (48)	65 (65)	0.114
Patients should have the right to be informed about all their rights at the time of admission.	78.3 (47)	91.7 (55)	78.3 (47)
Patients should have the right to obtain an adequate information about their clinical status in an understandable language.	83.3 (50)	69 (69)	0.044
The therapist should provide adequate information about the patients' treatment option in a manner appropriate to his/her clinical condition in an understandable language.	86.7 (52)	80 (80)	0.283
Patients should have the right to receive sufficient information about a rare complication of treatment in compliance with medical needs.	71.7 (43)	78 (78)	0.366
Patients should have the right to ask for explanation about the risks and complication of the therapeutic plan offered.	86.7 (52)	81 (81)	0.354
Patients should have the right to be informed about their chances for healing and recovery.	88.3 (53)	74 (74)	0.03
The therapist should introduce him/herself to the mentally ill patients.	73.3 (44)	68 (68)	0.476
The therapist should provide information about the professional role and responsibility of the medical team to the mentally ill patients.	80 (48)	66 (66)	0.58
Patients should be permitted to access their medical file.	16.7 (10)	77 (77)	0.0000
Patients should have the right to participate actively in all decisions and to have input in treatment planning.	55 (33)	64 (64)	0.259
Assessing the mental state of patients to determine the decision-making capacity of patients at every clinical encounter.	80 (48)	65 (65)	0.114
Patients have the right to be informed about error by the person who commits an error during service delivery.	33.3 (20)	83 (83)	0.000
Presence of a legal authority to change the decision taken by the service providers for the patient to preserve the patient's best interests.	88.3 (53)	51 (51)	0.000
Informed consent should be obtained about educational and research activities in which the patients will be present.	66.7 (40)	74 (74)	0.321
Patients should have the right to withdraw from treatment against the advice of the medical team at any time with or without cause.	38.3 (23)	73 (73)	0.000
The patient should have the right to refuse the recommended treatment.	48.3 (29)	73 (73)	0.000
The patient should have the right to leave the hospital against the advice of the medical team with personal consent.	48.3 (29)	74 (74)	0.001
In case of violation of rights, the patient should have the right to be able to use complaints and lawsuits and any application within the framework of legislation.	78.3 (47)	75 (75)	0.631
The staff should have the right to force hospitalization against a person’s will if the person is violent or and is a source of danger to others.	80 (48)	76 (76)	0.557
The staff should have the right to force hospitalization against a person’s will if the person is psychotic and thereby is not able to take care of his/her needs and neglects him/herself physically or mentally.	83.3 (50)	81 (81)	0.711
The staff should have the right to force hospitalization against a person’s will after a serious suicide attempt or if the person makes suicidal threats that seem serious.	88.3 (53)	81 (81)	0.223
There are circumstances under which the staff should have the right to confine the patients in a locked room.	88.3 (53)	51 (51)	0.000
There are circumstances under which the staff should have the right to use physical coercion, such as strapping, straitjackets, forced feeding, or injections.	80 (48)	52 (52)	0.000
The staff should have the right to ask patients to obey the ward rules, such as time of sleeping and awakening, eating, proper behavior, and rules about outings.	85 (51)	72 (72)	0.59
The therapist should have the right to pass on information in response to a query of the courts and legal authorities about the patient, his/her hospitalization and mental condition, even without the patient’s consent.	63.3 (38)	77 (77)	0.63
The therapist should be allowed to give information about the patient’s hospitalization and mental condition to his or her mate, even without the patient’s consent.	65 (39)	71 (71)	0.43
The healthcare provider should not disclose any information about the patient to the employers without the patient’s consent.	63.3 (38)	77 (77)	0.63
